# A Hybrid Information Reconciliation Method for Physical Layer Key Generation

**DOI:** 10.3390/e21070688

**Published:** 2019-07-14

**Authors:** Guyue Li, Zheying Zhang, Yi Yu, Aiqun Hu

**Affiliations:** 1 School of Cyber Science and Engineer, Southeast University, Nanjing 210096, China; 2 Institut Supérieur d’Electronique de Paris, 75006 Paris, France; 3 Conservatoire National des Arts et Métiers, 75003 Paris, France

**Keywords:** information reconciliation, comprehensive reconciliation efficiency, physical layer security, secret key generation

## Abstract

Physical layer key generation (PKG) has become a research focus as it solves the key distribution problem, which is difficult in traditional cryptographic mechanisms. Information reconciliation is a critical process in PKG to obtain symmetric keys. Various reconciliation schemes have been proposed, including the error detection protocol-based approach (EDPA) and error correction code-based approach (ECCA). Both EDPA and ECCA have advantages and drawbacks, regarding information leakage, interaction delay, and computation complexity. In this paper, we choose the BBBSS protocol from EDPA and BCH code from ECCA as a case study, analyzing their comprehensive efficiency performance versus pass number and bit disagreement ratio (BDR), respectively. Next, we integrate the strength of the two to design a new hybrid information reconciliation protocol (HIRP). The design of HIRP consists of three main phases, i.e., training, table lookup, and testing. To comprehensively evaluate the reconciliation schemes, we propose a novel efficiency metric to achieve a balance of corrected bits, information leakage, time delay, and computation time, which represents the effectively corrected bits per unit time. The simulation results show that our proposed method outperforms other reconciliation schemes to improve the comprehensive reconciliation efficiency. The average improvement in efficiency is 2.48 and 22.36 times over the BBBSS and BCH code, respectively, when the range of the BDR is from 0.5% to 11.5%. Compared to the BBBSS protocol and the BCH code, HIRP lies at a mid-level in terms of information leakage and computation time cost. Besides, with the lowest time delay cost, HIRP reaches the highest reconciliation efficiency.

## 1. Introduction

Wireless communication is ubiquitous in our daily life, and it is expected to support extremely high data rates and radically new applications in the foreseeable future. Meanwhile, wireless transmission is vulnerable to eavesdropping attacks due to the broadcast nature of the wireless medium. Therefore, safeguarding data transmission is given the top priority in the development of next-generation wireless networks [1,2]. A paradigmatic problem of securing data transmission is the key distribution. Traditional public key cryptography techniques are widely used in existing communication networks. However, they require a public key infrastructure and are computationally intense, and thus encounter key distribution and management difficulties in the limited-resource mobile networks. Furthermore, with the advent of quantum computers capable of rapidly performing a complex and massive factorization, the traditional cryptography mechanism based on computation complexity is no longer reliable.

Recently, physical layer key generation (PKG) has been emerging as a supplement to the upper layer key distribution method [2]. The underlying idea of it lies in the use of channel reciprocity and the uncertainty of multipath characteristics to encrypt the transmitted information in order to solve the problem of symmetric secret key distribution [3,4]. Besides, the spatial variation prevents eavesdroppers from observing the same randomness as legitimate users, for a sufficiently large distance between them.

Although the uplink and downlink channels are reciprocal, measurements of radio channels are not the same, due to the differences originating from additive noise, transceiver hardware, and time delay in time division duplex (TDD) systems [5]. However, the objective of PKG is to generate a pair of strict identical symmetric keys. Even one bit of difference would lead to decryption failure due to the avalanche effect. To address this issue, information reconciliation is exploited to detect and correct errors in the preliminary key material between the two communicating parties [6].

Several information reconciliation approaches have been proposed in previous work. Generally, these approaches can be classified into two categories, i.e., error detection protocol-based approaches (EDPAs) and error correction code-based approaches (ECCAs). EDPAs, such as BBBSS [7], Cascade [8], and Winnow [9], use multiround interactive parity checks to eliminate mismatches. In EDPAs, Alice divides the preliminary key material into small blocks and sends the parity information of each block to Bob. Bob divides his key material in the same way, computes parity check bits, and checks for mismatches. For each mismatch, Bob performs a binary search on the block to find a correction vector, which may fix the errors. These steps are iterated a number of times to ensure a high probability of success. Bennett et al. proposed BBBSS on the permute-and-bisect method in the first implementation of quantum key distribution (QKD) [7]. To reduce information leakage, Brassard and Salvail proposed an improved scheme called Cascade in [8]. Cascade uses the information in the preceding passes to correct errors in the parity check of the current pass. The parity check in bisect is also replaced with an MD5 hash [10] and by a cyclic redundancy check (CRC) [11] to further reduce the information leakage of Cascade. A more efficient implementation of Cascade, using some inherent information already available in the protocol, exactly known bits, and already known parities, was proposed in [12]. However, these approaches lead to more interactions since parallelization becomes much more challenging. Winnow is another interactive reconciliation protocol introduced by Buttler et al. in [9]. Different from BBBSS and Cascade, Winnow resolves the errors in the block using a syndrome calculation with Hamming codes. Parity bits and syndromes can be calculated and exchanged in parallel, making the protocol less interactive than Cascade. However, Winnow can introduce new errors if the error count per block is more than two. A modified one-way error reconciliation protocol that employed a mixed Hamming code concatenation scheme was proposed to study the relationship between the error correction capability and the key generation efficiency in [13].

On the other hand, it has been shown that reconciliation can be deemed as a special case of channel coding [6]. Therefore, existing coded modulation techniques can be adapted for reconciliation. ECCA corrects the mismatches using error correction codes such as Hamming, BCH codes, and low density parity check (LDPC) codes [6,13,14,15]. Alice and Bob divide the preliminary key material into vectors. By utilizing the error correction code, Alice calculates and sends the parity check bits to Bob. Bob applies the corresponding decoder, whereby the required code word is composed of Bob’s information vector and the received parity bits. If the number of bit disagreements is smaller than the error correcting capability, synchronized key material is guaranteed. Only one-round interaction is required in ECCA. In [13], a reconciliation protocol is proposed that is based on a mixed combination of Hamming syndrome concatenation. A reconciliation scheme, which uses syndromes of a BCH code for error correction and a one-bit feedback to report a successful decoding, is studied in [16]. In [17], the authors proposed using Turbo codes for reconciliation purposes. An information reconciliation protocol based on a rate-compatible construction of LDPC codes was proposed in [18]. However, the information leakage and computation complexity are generally increased in ECCA.

In existing work, reconciliation efficiency is the most commonly used evaluation metric, which is inversely proportional to leakage bit rate (LBR). However, rare work takes into account the interaction delay and computation complexity. But these factors may affect reconciliation efficiency greatly in some specific scenarios. For example, heavy interaction is unbearable in long-distance satellite communications. In Internet of Things (IoT) networks with limited resources, computation complexity has to be considered. Furthermore, both EDPA and ECCA have their pros and cons. There is still a gap regarding how to integrate their strengths to improve the reconciliation efficiency. To address these problems, this paper carries out a comprehensive and theoretical study on the information reconciliation schemes to establish highly efficient identical secret keys. Our contributions are as follows.

- A comprehensive reconciliation evaluation metric is proposed, taking consideration of LBR, interaction delay, and computation overhead. The metric represents the effective corrected bit number per unit time. The calculation expression of the metric is derived in this paper.
- The characteristics of BBBSS and BCH are analyzed from the perspective of the proposed new metric. Combining advantages of the two together, a new hybrid information reconciliation protocol (HIRP) is proposed. The detailed realization steps of HIRP are presented, including training, table lookup, and testing.
- The simulation results verify the theoretical analysis of both BBBSS and BCH. Monte Carlo simulations validate that the proposed HIRP outperforms the other two approaches to provide a more efficient information reconciliation in PKG.

The rest of the paper is organized as follows. Section 2 introduces the system model, the secret key generation process, the general information reconciliation model, and the problem studied in this paper. Section 4 provides a comprehensive reconciliation efficiency metric and presents the calculation expression for each factor. Section 5 proposes a new hybrid information reconciliation protocol (HIRP) and designs the realization algorithms. Section 6 presents the simulation results, and Section 7 concludes the paper.

## 2. System Model

### 2.1. General System Model

We consider a general Single Input single output single eavesdropper (SISOSE) model. All the users are equipped with a single antenna. Alice and Bob are two distinct legitimate users with a distance between each other of *d* meters. The communication system works at a frequency of $f_{c}$ GHz with a bandwidth of *B* Hz. The data transmission rate is then *B* bits per second (bps). Alice and Bob intend to extract secret keys from their channel characteristics to protect the data transmission. Key generation requires a temporally dynamic channel, and the channel variation can be introduced by the movement of users and/or surrounding objects [19].

Eve is a passive eavesdropper located more than the coherence distance from both Alice and Bob. According to the definition of coherence distance, the coherence distance at a carrier frequency of 2.4 GHz is 6.25 cm. Therefore, we assume that Eve experiences a fading channel independent of that of Alice and Bob. Despite this, Eve knows the whole communication protocols, the pilots, and all the information transmitted over the public channels between Alice and Bob.

The notations used in this paper, and their definitions, are summarized in Table 1.

### 2.2. Secret Key Generation Process

**Figure 1:**
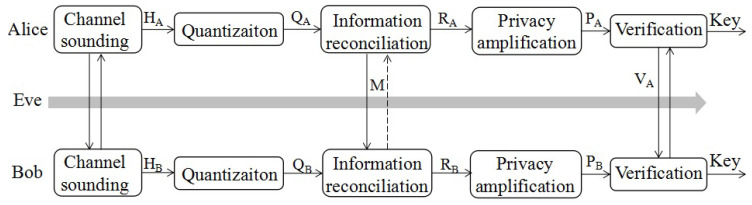
Physical-layer-based secret key generation process.

Considering the $t_{i}\in \left\{1,2,\cdots \right\}$-th round of secret key generation, Alice and Bob generate secret keys during a period of time *T*, as shown in Figure 1, which includes four main steps: channel sounding, quantization, information reconciliation, and privacy amplification. At first, Alice and Bob estimate their channel characteristics through channel sounding, i.e., sending pilots to each other. Eve may also estimate her channel to Alice or Bob. For simplicity, Eve’s channel is referred to as that between Eve and Bob in this paper. Denote the channel characteristics estimated during *T* for the user u∈{A,B,E} as $H_{u}$ with length $L_{H}$, where A, B, and E represent Alice, Bob, and Eve, respectively. Secondly, the user *u* maps the input values from $H_{u}$ into output values in a bit sequence set through quantization, e.g., channel quantization with guardband (CQG) used in [20]. The quantized bit sequence is represented as $Q_{u}$ with length $L_{Q}$.

Until now, there has existed unavoidable bit disagreements between $Q_{A}$ and $Q_{B}$, caused by time delay in TDD systems, hardware differences, and noise [21]. Although some preprocessing approaches, e.g, principal component analysis (PCA) [20], are applied, the bit disagreements are not fully eliminated. However, even a bit difference in a secret key will trigger an avalanche effect, leading to complete decryption failure. To deal with this problem, Alice and Bob correct the bit disagreements of $Q_{u}$ through information reconciliation, and the corrected bit sequence is denoted by $R_{u}$ with length $L_{R}=L_{Q}$. Totally, the $L_{M}$ bits of parity information *M* are transmitted during the reconciliation process. The dashed line in Figure 1 shows that the communication may either be bidirectional or one way. Unfortunately, *M* is also leaked to Eve as she knows all the information transmitted through public channels. According to the leftover hash lemma, $L_{M}$ bits arbitrarily chosen for $R_{u}$ are discarded to guarantee key security during the privacy amplification step. For example, when $L_{M}=40$ and $L_{R}=168$, a simple realization method is to map the 168-bit corrected sequence $R_{u}$ to a 128-bit random sequence $P_{u}$ through an MD5 hash function. Finally, the key consistency is verified by sending a simple hash value $V_{u}$ of $P_{u}$ from one to another. When the hash value is identical, the $t_{i}$-th round of secret key generation is successful. Otherwise, the $t_{i}$-th round of secret key generation fails, and the $P_{u}$ is discarded.

### 2.3. A General Model for Information Reconciliation

Various approaches, including EDPA and ECCA, are proposed for information reconciliation. In this section, we establish a general model for them. During the information reconciliation step, Alice communicates with Bob over public channels for *K* passes. All information transmitted over public channels is assumed to be error-free.

An EDPA, such as BBBSS, has J(k),k=1,2,⋯,K rounds of back-and-forth interactions for the *k*-th pass. In each round of interaction, Alice first sends the parity information to Bob, then Bob feeds back the information about error position to Alice. On the other hand, an ECCA, such as BCH codes, generally only has one pass and one round of communication, i.e., K=1 and J=1. It is because the error correct code has error propagation when the error number is beyond its error correcting capability. Therefore, it is inefficient for ECCA to gradually reduce bit error numbers through multiple passes or rounds. Besides, ECCA is a one-way communication in which Alice sends a syndrome to Bob but Bob does not provide feedback. Bob uses the syndrome to correct his channel observation through decoding algorithms, e.g., the Viterbi algorithm.

**Figure 2:**
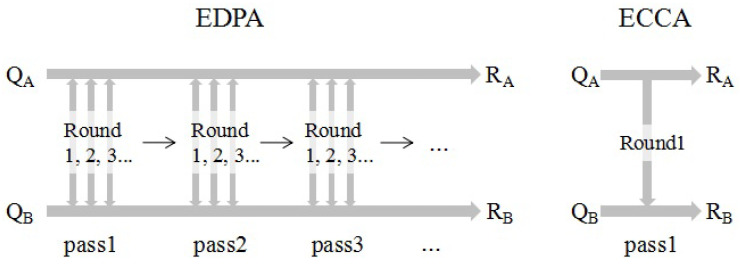
Illustration of information reconciliation process for the error detection protocol-based approach (EDPA) and error correction code-based approach (ECCA).

Figure 2 illustrates the information reconciliation process for both EDPA and ECCA. During the *k*-th pass of communication, Alice and Bob divide $Q_{u}$ into $N_{G}\left(k\right)$ groups with group length $L_{RG}\left(k\right)$. Denote $N_{aGE}$ as the estimated average number of error bits in one group. For EDPA, group length $L_{RG}\left(k\right)$ is designed to guarantee that each group has about one error, i.e., $N_{aGE}=1$. During the first round of communication (J(k)=1), Alice sends the parity of each group to Bob, and Bob feeds back the indexes of wrong groups. A group is defined as an error group if the parity information of Alice and Bob is different. Then, for each wrong group, J(k)−1 rounds of bisect error-correcting are applied to find the position of error bit. As for ECCA, group length $L_{RG}$ depends mainly on the affordable decoding complexity of Bob. In the affordable range, the larger the $L_{RG}$, the more accurate the $N_{aGE}$. Therefore, $L_{RG}$ is usually set as the largest affordable length. Each group may have more than one error in this case. According to the signal-to-noise ratio (SNR), it is estimated that the ratio of $N_{aGE}$ to $L_{RG}$ matches the coarse bit disagreement ratio (BDR) estimation of $Q_{u}$. Then, ECCA chooses the error correction code $C(n_{c},k_{c},t_{c})$, where $n_{c}$, $k_{c}$ and $t_{c}$ are the code length, message length, and error correcting number, respectively. Code $C(n_{c},k_{c},t_{c})$ satisfies that the message bit length $k_{c}=L_{RG}$ and the correction error number $t_{c}\geq N_{aGE}$. Next, Alice divides $Q_{A}$ into groups and sends all groups of syndromes to Bob. According to the syndromes, Bob corrects the inconsistent bits in $Q_{B}$ using decoding algorithms.

## 3. Problem Statement

Information reconciliation approaches are mainly categorized as EDPA or ECCA, and each has its advantages and drawbacks. The downside to EDPA is that it needs multiple passes and multiple rounds of back-and-forth interactions, as shown in Figure 2. When Alice is far away from Bob, it causes a very large interaction delay and communication overhead. Furthermore, the efficiency of EDPA decreases with the increase in pass number. The proof is provided in Section 3. On the plus side, EDPA just uses bisect error-correcting, which consumes less computation and leaves less leakage of information.

Conversely, ECCA only has one pass, one round, and one-way communication. Obviously, the interaction delay and communication overhead are significantly reduced. The negative side of ECCA is that it has expensive computation overhead and large information leakage, especially for low SNR scenarios. If $L_{RG}$ is small, the estimate of $N_{aGE}$ is inaccurate, which may lead to error propagation. Instead, if $L_{RG}$ is large, the decoding complexity is high. Even worse, information leakage increases rapidly with the rise of $N_{aGE}$ for ECCA. Table 2 summarizes the features of EDPA and ECCA.

Since both EDPA and ECCA have their pros and cons, this raises a natural question: “How to comprehensively evaluate the performance of an information reconciliation approach?” Existing work only considers one or two indicators of performance, e.g., information leakage, leaking the evaluations of interaction time, complexity, etc. The subsequent problem is: “Is it possible to integrate the strengths of both EDPA and ECCA to design a new reconciliation approach that makes a trade-off of all these performance indicators?” To address this problem, we first propose a comprehensive metric to evaluate the efficiency of reconciliation approaches. Next, we discuss the performance of EDPA and ECCA, respectively. In this paper, we choose BBBSS of EDPA and BCH code of ECCA as a case study. Under the guidance of the new metric, we then design a new approach named HIRP to achieve good efficiency.

## 4. A Comprehensive Information Reconciliation Evaluation Metric

In this section, we propose a comprehensive reconciliation efficiency metric, taking consideration of corrected bits, information leakage, interaction delay, and computation time.

### 4.1. Information Leakage

Information reconciliation poses a security threat as eavesdroppers can infer keys from the interacted information. Therefore, the information leakage should be considered when evaluating an information reconciliation scheme.

**Definition** **1.** *Denote η as the information leakage ratio, which is defined by*

(1) $$\eta =\frac{I(R_{A},M)}{L_{R}},$$

*where $R_{A}$ is the reconciled key with length $L_{R}$, M is information disclosed during interaction, and $I(R_{A},M)$ is the mutual information between them, which represents the information that eavesdroppers can obtain about the key. To guarantee the security of the final key, at least η proportion of the reconciled keys should be wiped off in the privacy amplification step.*

**Remark** **1.** *Denote $L_{M}$ as the length of M and ε as the BDR between $Q_{A}$ and $Q_{B}$, then the lower bound and upper bound of η are derived as*

(2) $$h\left(\varepsilon \right)\frac{L_{Q}}{L_{R}}\leq \eta \leq \frac{L_{M}}{L_{R}},$$

*where h(ε) is the entropy of ε with*

(3) h(ε)=−ε·logε−(1−ε)·log(1−ε).

*The lower bound of η represents the minimum amount of interaction information per bit for $Q_{A}$ and $Q_{B}$ to obtain identical keys. Since $L_{M}$ is the length of M,  then the maximum disclosed bits is $L_{M}$. When M has a linear relationship with $Q_{A}$, the disclosed bits is $L_{M}$. Otherwise, it is less than $L_{M}$ due to the increased ambiguity caused by nonlinearity. Therefore, the upper bound of η is $L_{M}/L_{R}$.*

*In this paper, we calculate the information leakage ratio through its upper bound with $\eta =\frac{L_{M}}{L_{R}}$ for security purposes.*

### 4.2. Interaction Delay

The interaction delay represents the time spent on exchanging information *M*. It can become significant in EDPA, which has multiround interactions. Denote $T_{delay}$ as the interaction delay, which includes two parts, i.e., the data transmission time and the propagation time. Then, $T_{delay}$ is calculated as

(4) $$T_{delay}=T_{data}+T_{prop}=\frac{L_{M}}{B}+2\sum_{k=1}^{K}J\left(k\right)\left(\frac{d}{c}+T_{0}\right),$$

where *B* is the system bandwidth, $\sum_{k=1}^{K}J\left(k\right)$ is the number of back-and-forth interactions, *d* is the transmission distance, *c* is the velocity of light, and $T_{0}$ is the communication overhead in every back-and-forth interaction.

As derived in (1), $L_{M}\geq h\left(\varepsilon \right)L_{Q}$, and thus

(5) $$T_{delay}\geq \frac{h\left(\varepsilon \right)L_{Q}}{B}+2\sum_{k=1}^{K}J\left(k\right)\left(\frac{d}{c}+T_{0}\right).$$

The latter term rises with the increase of $\sum_{k=1}^{K}J\left(k\right)$. Besides, in long-distance communications, such as satellite communications, the latter term becomes the dominant factor for long interaction delays. Therefore, the number of information interactions should be lowered to reduce the delay and communication overhead.

### 4.3. Computation Time

In some resource-constrained systems, the performance of error-correcting schemes may be constrained since decoding algorithms require multiple round iterations. Therefore, computation complexity, which is characterized by the computation time $T_{c}$, should be taken into account. Denote $T_{c}$ as

(6) $$T_{comp}=t_{c}\cdot N_{eqAdd},$$

where $t_{c}$ is the time cost of an “equivalent addition” and $N_{eqAdd}$ represents the number of equivalent additions. The required mathematical and logical operations can be viewed as multiples of “equivalent addition” due to current digital signal processor (DSP) specifications in [22]. In Table 3, computation operations are normalized to 5. $T_{comp}$ is determined by the BDR of initial keys, group size, and decoding complexity. The higher the BDR is, the longer the computation time is. Generally, ECCA has a much heavier computation cost than  EDPA.

### 4.4. Effective Reconciliation Rate ξ

To achieve a balance in the above factors, we propose a novel comprehensive metric ξ, which is called the effective reconciliation rate, to evaluate the performance. The definition of ξ is given by

(7) $$\xi =\frac{\left(1-\eta \right)N_{corr}}{T_{delay}+T_{comp}},$$

where $N_{corr}$ denotes the number of corrected inconsistent bits. Actually, ξ represents the effective corrected bit number per unit time. Therefore, it reflects the efficiency of an information reconciliation approach. There is a negative correlation between ξ and information leakage η, and interaction delay $T_{delay}$ and computation time $T_{comp}$. Reducing the value of η, $T_{delay}$, and $T_{comp}$ contributes to the improvement of ξ. The higher the ξ is, the more efficient the information reconciliation approach is.

## 5. A Hybrid Information Reconciliation Protocol

In this section, we first review the characteristics of BBBSS and BCH from the perspective of the new metric ξ. Combining the advantages of both, we propose a new approach named HIRP, which aims to improve the comprehensive reconciliation efficiency.

### 5.1. BBBSS

**Figure 3:**
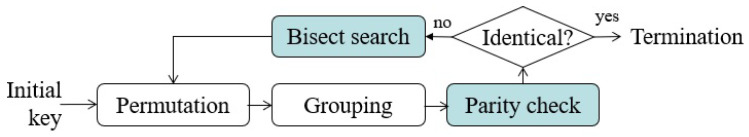
The flow chart of BBBSS.

The BBBSS protocol uses permutation-and-bisect block to remove the discrepancies [23]. Define one pass of bisect and permutation block correction as one pass of BP. Figure 3 illustrates the flow chart of BBBSS, in which solid blocks contain information interactions. Permutation distributes disagreements randomly and then groups key strings into blocks using estimated BDR. The block length is recommended as $L_{RG}\left(k\right)=0.73/\varepsilon (k)$, where ε(k) is the BDR for the *k*-th pass. Then Alice and Bob interact the parity check of each block to find out error blocks and apply bisect error correcting to correct disagreements. Since this method couldn’t detect the block that has an even number of disagreements, multiple passes of BPs are required. The pass iteration terminates when the parity check of all the blocks are identical.

We further define the efficiency metric in the *k*-th pass as

(8) $$\xi \left(k\right)=\frac{\left(1-\eta^{\left(k\right)}\right)N_{corr}^{\left(k\right)}}{T_{delay}^{\left(k\right)}+T_{comp}^{\left(k\right)}}.$$

The information leakage satisfies that

(9) $$\eta^{\left(k\right)}=\frac{L_{M}\left(k\right)}{L_{R}}=\frac{N_{G}\left(k\right)+N_{EG}^{\left(k\right)}\left[J\left(k\right)-1\right]}{L_{R}},$$

where $N_{EG}^{\left(k\right)}=N_{corr}^{\left(k\right)}$ indicates the number of error groups for the *k*-th pass and $J\left(k\right)=\left\lceil \log_{2}L_{RG}\left(k\right)\right\rceil +1$ is the number of back-and-forth interactions for the *k*-th pass. Except for the first round of finding the error groups, it needs additional $\left\lceil \log_{2}L_{RG}\left(k\right)\right\rceil$ rounds to find the error position.

The time delay satisfies that

(10) $$T_{delay}^{\left(k\right)}=\frac{L_{M}\left(k\right)}{B}+2\sum_{k=1}^{K}J\left(k\right)\left(\frac{d}{c}+T_{0}\right).$$

The computation time $T_{comp}$ has a linear growth with $L_{M}\left(k\right)$.

In general, with the increase of pass number *k*, the group number $N_{G}\left(k\right)$ and corrected bits $N_{corr}^{\left(k\right)}$ decline. However, the group length $L_{RG}\left(k\right)$ increases, and thus the interaction number J(k) increases. As stated in Section 4.2, the latter term in the time delay plays a dominant role. When *k* is small, one round of interaction is more efficient as it processes parity information for multiple groups in parallel. However, when *k* is large, even one error group may need a round of interaction, which causes low efficiency. With $N_{corr}^{\left(k\right)}$ and $T_{delay}^{\left(k\right)}$ playing dominant roles, ξ decreases with the increase in pass number, which is also verified in the simulations of Section 6. To sum up, BBBSS has high efficiency at the first several passes and then becomes less efficient in subsequent passes.

### 5.2. BCH

BCH code with $C(n_{c},k_{c},t_{c})$ has only one pass of interaction. Since each group has the same code, the ξ in one group is equal to the whole ξ. Then the leakage rate satisfies that

(11) $$\eta =\frac{n_{c}-k_{c}}{k_{c}}.$$

When $C(n_{c},k_{c},t_{c})$ is capable of correcting all of the errors, then $N_{corr}=t_{c}$. The time delay is

(12) $$T_{delay}=\frac{n_{c}-k_{c}}{B}+\frac{d}{c}+T_{0}.$$

At last, $T_{comp}$ rises with the increase of $t_{c}$.

The metric ξ is

(13) $$\xi =\frac{\left(1-\frac{n_{c}-k_{c}}{k_{c}}\right)t_{c}}{\left(\frac{n_{c}-k_{c}}{B}+\frac{d}{c}+T_{0}\right)+T_{comp}\left(t_{c}\right)}.$$

To correct $t_{c}$ errors, it has to be satisfied that $n_{c}-k_{c}\geq 2t_{c}+1$. Assume that $n\approx k_{c}+\left(2t_{c}+1\right)$, then Equation (13) is approximated as

(14) $$\xi \approx \frac{\left(1-\frac{2t_{c}+1}{k_{c}}\right)t_{c}}{\left(\frac{2t_{c}+1}{B}+\frac{d}{c}+T_{0}\right)+T_{comp}\left(t_{c}\right)}.$$

In one group, $C(n_{c},k_{c},t_{c})$ satisfies that the message bit length $k_{c}=L_{RG}$ and the correction error number $t_{c}\geq N_{aGE}$. Thus, $\varepsilon \approx t_{c}/k_{c}$, and $k_{c}$ is a constant that mainly depends on the affordable decoding complexity of Bob. Thus, Equation (15) is further written as

(15) $$\xi \approx \frac{a_{1}\varepsilon^{2}+a_{2}\varepsilon }{a_{3}\varepsilon +T_{comp}\left(\varepsilon \right)+a_{4}},$$

where $a_{1}=-2k_{c}$, $a_{2}=k_{c}-1$, $a_{3}=\frac{2k_{c}}{B}$, $a_{4}=\frac{1}{B}+\frac{d}{c}+T_{0}$. $T_{comp}\left(\varepsilon \right)$ decreases monotonously along with increasing ε. Because $a_{1}<0$ and $a_{3}>0$, ξ decreases along with increasing ε generally. In summary, BCH is more efficient in low BDR regions.

### 5.3. The Algorithm of the Proposed HIRP

In the previous analysis, both BBBSS and BCH are efficient in some specific conditions. On the one hand, BBBSS is effective for the first few passes of BP, and its BDR is reduced down (about threefold) after each pass. On the other hand, BCH shows better efficiency at low BDR regions. Inspired by these, we propose a hybrid approach named HIRP to further improve the reconciliation efficiency, combining the virtues of both BBBSS and BCH.

**Figure 4:**
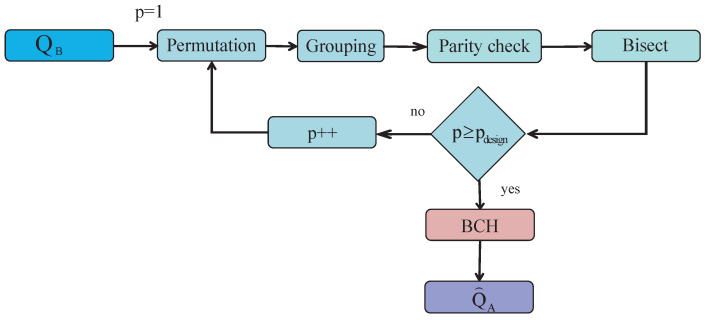
The flow chart of the hybrid information reconciliation protocol (HIRP).

Figure 4 illustrates the flow chart of HIRP. The core idea is that when the BDR is high, several passes are firstly exploited to reduce it to a low value, and then the few residual errors are further corrected by BCH, which is efficient in low BDR regions. Algorithm 1 gives the details of the realization steps of HIRP, which contains three main phases, i.e., training, table lookup, and testing.

**Algorithm 1** Algorithm of HIRP**Input:** training data: $Q_{A}^{Train}$ and testing data: $Q_{A}^{Test},Q_{B}^{Test}$       **Output:** Estimated testing data ${\hat{Q}}_{A}$ **Training phase:**
Add noise to $Q_{A}^{Train}$ to generate $Q_{B}^{Train}$ with different BDR ε.Traverse all possible BPs for different ε in range and calculate their efficiency respectively.Find the optimal pass number to maximize the efficiency ξ and draw Table 4.**Table lookup phase:**
Mark the locations of $p_{optimal}$ in Table 5.Find the threshold BDR $\varepsilon_{th}$.Calculate the designed pass number $p_{designed}$ and draw Table 6.**Testing phase:**
Estimate the ε of $Q_{A}^{Test}$ and $Q_{B}^{Test}$.Select $p_{designed}$ from Table 5 with the estimated BDR.Use the $H_{p}$ algorithm for reconciliation, which applies $p_{designed}$ passes of BPs firstly and then eliminates remaining disagreements by BCH codes.

**Figure 5:**
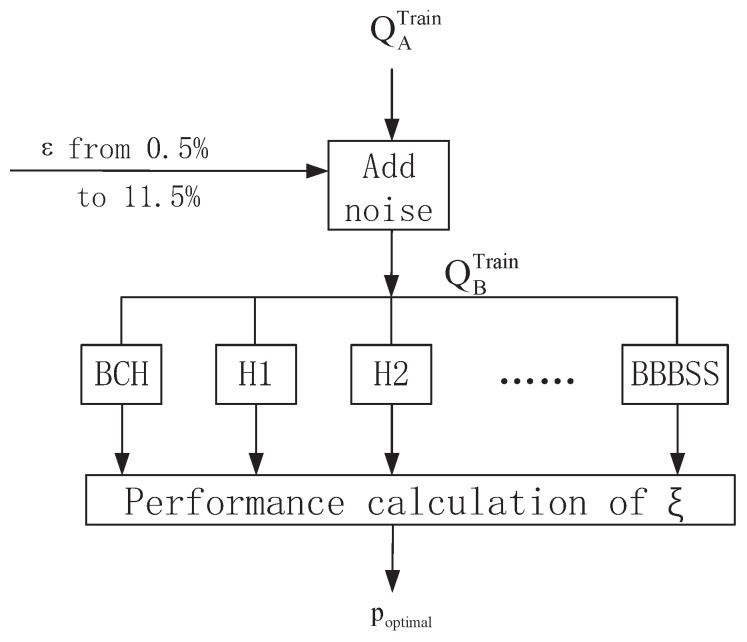
Block diagram of training phase.

Define $H_{p}$ as the HIRP approach with *p* passes of BP. When p=0, HIRP turns into BCH, and when *p* gets large enough, HIRP is equal to BBBSS. The parameter selection of *p* is critical to our proposed approach. In the training phase, we first collect the optimal *p* values that achieve the maximal ξ for a group of BDRs. Figure 5 shows a realization framework to find $p_{optimal}$. By adding artificial noise to training data $Q_{A}^{Train}$, we get the desired $Q_{B}^{Train}$ with BDR ε ranging from 0.5% to 11.5%. The collected results of $p_{optimal}$ versus ε are shown in Table 4.

Although when $p=p_{optimal}$, $H_{p}$ can achieve the highest ξ, the traversing method is complicated, and the cost is huge in practical applications. Besides, it is challenging to go through all the possible $H_{p}$s for every possible ε. To deal with the problem, we design a new table of $p_{design}$ versus ε with the combination of both Table 4 and Table 5. The element $\left(\varepsilon_{in},p,\varepsilon_{out}\right)$ in Table 5 represents the input BDR, pass number, and the corresponding output BDR. From Table 5, the BDR is reduced to roughly one third after every pass. After *p* passes, the BDR of output signals $\varepsilon_{out}$ satisfies that

(16) $$\varepsilon_{out}\approx \varepsilon {\left(\frac{1}{3}\right)}^{p}.$$

To simplify the process, $p_{designed}$ is calculated as the minimum value of *p*, that satisfies

(17) $$\varepsilon_{out}\approx \varepsilon {\left(\frac{1}{3}\right)}^{p}\leq \varepsilon_{th},$$

where the threshold $\varepsilon_{th}$ is set as the largest value of $\varepsilon_{out}$ among the marked elements. From Table 5, the threshold of our simulation is $\varepsilon_{th}=0.425$. According to the above rules, Table 6 gives the value of $p_{designed}$ versus different ε.

**Figure 6:**
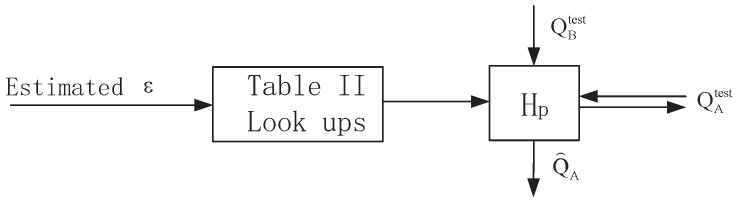
Block diagram of testing phase.

In the testing phase, Bob first estimates the ε. The coarse BDR estimation can be calculated according to the channel signal-to-noise ratio. After that, $Q_{A}$ and $Q_{B}$ are grouped with $len=0.73/\varepsilon_{coarse}$, which satisfies $N_{aGE}=1$. Then *A* and *B* interact the parity check of each block for a fine BDR estimation. Next, Bob selects the corresponding pass number $p_{designed}$ from Table 6. Finally, Bob conducts the algorithm of $H_{p}$ for information reconciliation and recovers the bit sequence of ${\hat{Q}}_{A}$. The block diagram of the testing phase is illustrated in Figure 6. The testing phase does not need sophisticated communication or a heavy computation cost. The additional operation is the table lookup, which is easy to realize in practice.

## 6. Simulations

In this section, we give some simulation results of the BBBSS, BCH, and our proposed HIRP scheme with $p_{optimal}$ and $p_{design}$ for comparison. The communication distance is set as d=5 KM, the communication bandwidth is B=4 MHz. The communication overhead in one interactive is set as $T_{0}=50$ ms for the consideration of packet loss.

**Figure 7:**
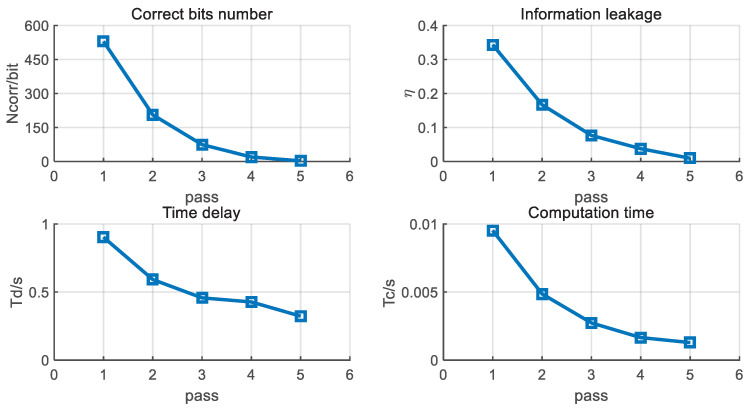
Individual performance of BBBSS per pass with ε=11%, d=5 KM, B=4 MHz, and $T_{0}=50$ ms.

**Figure 8:**
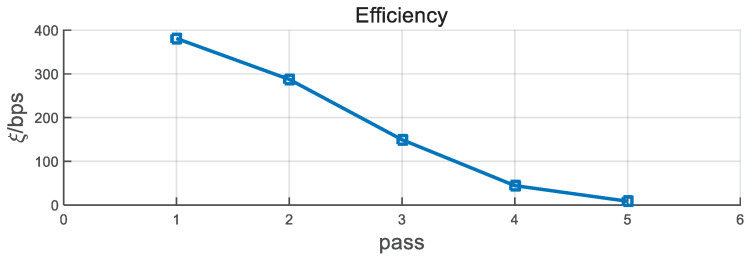
Efficiency performance of BBBSS per pass with ε=11%, d=5 KM, B=4 MHz, and $T_{0}=50$ ms.

First, we simulate the efficiency metrics of BBBSS in every individual pass. The results are given in Figure 7. Both the corrected bit number and information leakage ratio reduce with the increase in pass number. The interaction time delay rises at first and then goes down after the 4-th pass. This is caused by the fact that the group length $L_{RG}$ increases, while the number of error groups is not reduced significantly. Comprehensively, as shown in Figure 8, the metric ξ decreases with the increase in pass number, which means that BBBSS has a high efficiency at the first several passes. The simulation results coincide with the theoretical analysis in Section 5.1.

**Figure 9:**
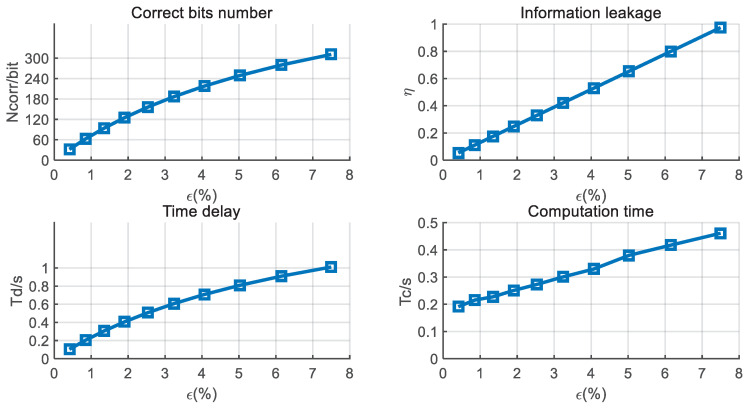
Individual performance of BCH versus BDR with d=5 KM, B=4 MHz, and $T_{0}=50$ ms.

**Figure 10:**
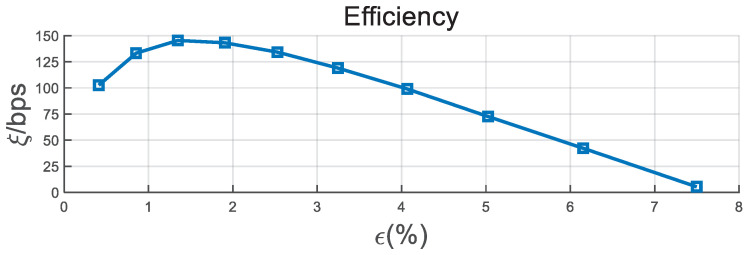
Efficiency performance of BCH versus BDR with d=5 KM, B=4 MHz, and $T_{0}=50$ ms.

We also simulate the individual performance of BCH for different BDRs in Figure 9. With the increase in the BDR, the information leakage ratio, the time delay, and the computation time show an upward trend. Therefore, the performance of ξ presents a general falling tendency, as shown in Figure 10. When the BDR is lower than 1.5%, the ξ has a slight increase. This is because the correct bit number has a significant rise with the increase in BDR.

**Figure 11:**
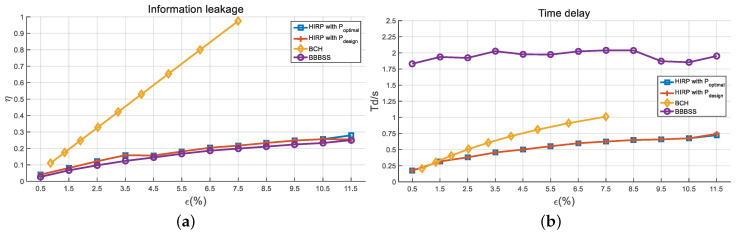
The comparison of (**a**) information leakage and (**b**) time delay. d=5 KM, B=4 MHz, and $T_{0}=50$ ms.

**Figure 12:**
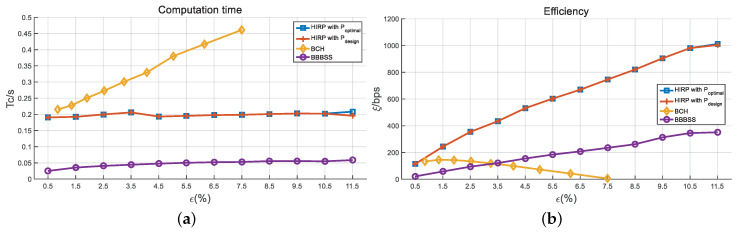
The comparison of (**a**) computation time and (**b**) efficiency. d=5 KM, B=4 MHz, and $T_{0}=50$ ms.

Next, we compare the performance in terms of the information leakage, the time delay, the computation time, and the comprehensive efficiency for various information reconciliation approaches including BBBSS, BCH, and HIRPs with optimal and designed pass numbers. Figure 11a shows the information leakage ratio versus BDRs. BCH has the highest information leakage ratio, which rises significantly with the increase in BDR. When the BDR is 7.5%, the BCH code is chosen as C(8191,4148,311), and the leakage ratio reaches 1. Therefore, we do not represent the BCH performance results for BDRs larger than 7.5%. The leakage ratio of the HIRPs is almost identical to that of BBBSS, and their growth is slow with BDR. Figure 11b represents the interaction time delay as a function of the BDRs. BBBSS has a longer interaction time compared with others. HIRPs have the shortest time delay and the slowest growth for BDRs above 1.5%. Figure 12a describes the computation time with respect to BDRs. The computation complexity rises significantly with the ramp-up of BDR. BCH has the longest computation time, which becomes significant in high BDR regions. BBBSS has the shortest computation time, and HIRPs have the middle one. In addition, the computation times of BBBSS and HIRPs rise slowly with the increase in BDR.

Figure 12b compares the efficiency ξ of different information reconciliation approaches. In low BDR regions, BCH has a better performance than BBBSS, while in high BDR regions, the opposite is true. It is observed that the ξ of HIRP outperforms that of both BBBSS and BCH along all BDR regions. It should be noted that when we only consider information leakage and computation time, HIRP seems to have no advantage compared to BBBSS. However, the time delay in Figure 11b shows that HIRP has a much lower time delay than BBBSS. The multipass interaction in the BBBSS protocol increases its time delay seriously. Therefore, the final comprehensive efficiency of HIRP is higher than that of BBBSS. In addition, HIRP with designed *p* has almost the same performance as HIRP with optimal *p*. The results verify the effectiveness of our proposed approach.

Table 7 shows the numerical improvement results of HIRP against BBBSS and BCH. According to Equation (7), the effective reconciliation rate ξ is inversely proportional to information leakage η, time delay $T_{delay}$, and computation time $T_{comp}$. Compared to BBBSS, the comprehensive efficiency ξ is improved 2.48 times, mainly due to the fact that HIRP declines $T_{delay}$ by 73% on average. Compared to BCH codes, HIRP declines η, $T_{delay}$, and $T_{comp}$, which results in the improvement of HIRP efficiency by an average of 22.36.

## 7. Conclusions

This paper examined the efficiency of information reconciliation approaches. We introduced a comprehensive reconciliation efficiency metric that considers the corrected bits, the interaction delay, and the computation time synthetically. Furthermore, we analyzed the characteristics of both BBBSS and BCH from the perspective of the metric. The efficiency of BBBSS decreases along with pass number, and BCH has low efficiency in high BDR regions. Inspired by this, we proposed a HIRP method that exploits certain passes of BP and then corrects the residual errors by BCH. The design of HIRP contains training, table lookup, and testing phases. The simulation results verified the effectiveness of our proposed HIRP approach. HIRP improves the comprehensive reconciliation efficiency 2.48 and 22.36 times compared with BBBSS and BCH, respectively. It makes a trade-off between individual performance indicators by achieving a median value of information leakage, interaction delay, and computation time. In the future, we plan to study the parameter design of HIRP from the theoretical point of view in some specific scenarios. In addition, we chose the BBBSS protocol of EDPA and the BCH code of ECCA as a case study in this paper. We plan to expand HIRP to a more general hybrid method considering more protocols and codes in EDPA and ECCA in our next step.

## Figures and Tables

**Table 1:** Notations used throughout the paper.

Notation	Definition
*B*	System bandwidth
*d*	Distance between Alice and Bob
$t_{i}$	The time round index of secret key generation
*T*	Period of each secret key generation round
*u*	User Alice, Bob, and Eve
$H_{u}$	Channel characteristics estimation of user u
$L_{H}$	Length of $H_{u}$
$Q_{u}$	Quantized bit sequence of *H* of user u
$L_{Q}$	Length of $Q_{u}$
$R_{u}$	Corrected bit sequence of *Q* of user u
$L_{R}$	Length of $R_{u}$
$P_{u}$	Bit sequence of *R* after privacy amplification of user u
$V_{u}$	Verification hash value of user u
*M*	Interactive information leaked during reconciliation
$L_{M}$	Length of *M*
*K*	Interaction passes in reconciliation
J(k)	Number of back-and-forth interaction rounds for the *k*-th pass
$N_{G}\left(k\right)$	Number of groups for the *k*-th pass
$L_{RG}\left(k\right)$	Length of each group for the *k*-th pass
$N_{aGE}$	Average number of error bits in one group
$C(n_{c},k_{c},t_{c})$	Error correction code,
	message length $k_{c}$ and error correcting bits $t_{c}$
ξ	Effective corrected bit number per unit time
η	Information leakage rate
$T_{delay}$	Time delay caused by interaction in reconciliation
$T_{comp}$	Computation time in reconciliation
$N_{corr}$	Corrected disagreement bit number
$N_{eqAdd}$	Number of “addition”
$N_{EG}$	Number of existing disagreement block detected by parity check

**Table 2:** Features of EDPA and ECCA.

Features	Interaction	Complexity	Leakage
EDPA	High	Low	low
ECCA	Low	High	High

**Table 3:** Equivalent addition conversion table.

Addition/Subtraction:	1	Division by 2:	1	Max/Min(2 arguments):	2
(±1)·Multiplication:	1	Table lookups:	6	Compare:	1

**Table 4:** Optimal p for different bit disagreement ratios (BDRs).

ε(%)	0.5	1.5	2.5	3.5	4.5	5.5	6.5	7.5	8.5	9.5	10.5	11.5
$p_{optimal}$	1	2	2	2	3	3	3	3	3	3	3	3

**Table 5:** Output BDR after p passes of bisect and permutation (BP).

	*p*	1	2	3	4	5	6
ε(%)							
0.5		0.184	0.060	0.016	0.003	0.000	0.000
1.5		0.564	0.186	0.049	0.009	0.001	0.000
2.5		0.944	0.312	0.082	0.015	0.002	0.000
3.5		1.304	0.425	0.108	0.018	0.002	0.000
4.5		1.699	0.559	0.142	0.024	0.002	0.000
5.5		2.065	0.672	0.170	0.028	0.002	0.000
6.5		2.438	0.789	0.196	0.030	0.002	0.000
7.5		2.721	0.862	0.205	0.030	0.002	0.000
8.5		3.093	0.988	0.240	0.035	0.002	0.000
9.5		3.405	1.068	0.249	0.034	0.002	0.000
10.5		3.635	1.084	0.236	0.029	0.001	0.000
11.5		4.187	1.293	0.303	0.042	0.002	0.000

**Table 6:** Designed p for different BDR.

ε(%)	0.5	1.5	2.5	3.5	4.5	5.5	6.5	7.5	8.5	9.5	10.5	11.5
$p_{designed}$	1	2	2	2	3	3	3	3	3	3	3	4

**Table 7:** Improvement of HIRP reconciliation factors.

	Compared to BBBSS				Compared to BCH Codes			
BDR	ξ	η	$T_{\mathrm{delay}}$	$T_{\mathrm{comp}}$	ξ	η	$T_{\mathrm{delay}}$	$T_{\mathrm{comp}}$
0.50%	4.35	0.50	−0.90	6.61	0.07	−0.37	0.40	−0.16
6.50%	2.22	0.10	−0.70	2.80	19.41	−0.76	−0.36	−0.53
Average	2.48	0.17	−0.73	3.38	22.36	−0.66	−0.20	−0.37
